# Silencing NKG2D ligand-targeting miRNAs enhances natural killer cell-mediated cytotoxicity in breast cancer

**DOI:** 10.1038/cddis.2017.158

**Published:** 2017-04-06

**Authors:** Jiaying Shen, Jie Pan, Chengyong Du, Wengong Si, Minya Yao, Liang Xu, Huilin Zheng, Mingjie Xu, Danni Chen, Shu Wang, Peifen Fu, Weimin Fan

**Affiliations:** 1 Program of Cancer Innovative Therapeutics, Division of Hepatobiliary and Pancreatic Surgery, Department of Surgery, The First Affiliated Hospital, College of Medicine, Zhejiang University, 79 Qingchun Road, Hangzhou 310000, China; 2 Department of Breast Surgery, The First Affiliated Hospital, College of Medicine, Zhejiang University, 79 Qingchun Road, Hangzhou, 310003, China; 3 Clinical Research Center, The First Affiliated Hospital, College of Medicine, Zhejiang University, 79 Qingchun Road, Hangzhou, 310003, China; 4 Department of Biological Sciences, National University of Singapore, 117543, Singapore; 5 Department of Pathology and Laboratory Medicine, Medical University of South Carolina, Charleston, SC 29425, USA

## Abstract

NKG2D is one of the major activating receptors of natural killer (NK) cells and binds to several ligands (NKG2DLs). NKG2DLs are expressed on malignant cells and sensitize them to early elimination by cytotoxic lymphocytes. We investigated the clinical importance of NKG2DLs and the mechanism of NKG2DL regulation in breast cancer (BC). Among the NKG2DLs MICA/B and ULBP1/2/3, the expression levels of MICA/B in BC tissues were inversely associated with the Tumor Node Metastasis stage. We first found that the high expression of MICB, but not MICA, was an independent prognostic factor for overall survival in patients with BC. Investigation into the mechanism revealed that a group of microRNAs (miRNAs) belonging to the miR-17-92 cluster, especially miR-20a, decreased the expression of ULBP2 and MICA/B. These miRNAs downregulated the expression of MICA/B by targeting the MICA/B 3'-untranslated region and downregulated ULBP2 by inhibiting the MAPK/ERK signaling pathway. Functional analysis showed that the silencing of NKG2DL-targeting miRNAs in BC cells increased NK cell-mediated cytotoxicity *in vitro* and inhibited immune escape *in vivo*. In addition, histone deacetylase inhibitors (HDACis) increased NKG2DL expression in BC cells by inhibiting members of the miR-17-92 cluster. Thus, targeting miRNAs with antisense inhibitors or HDACis may represent a novel approach for increasing the immunogenicity of BC.

In 2012, breast cancer (BC) accounted for 25% of all cancer cases worldwide and 15% of all cancer deaths among females.^1^ Late-stage BC is often associated with an immunosuppressive microenvironment and lacks of antitumor immune responses. Natural killer (NK) cells mediate direct cytotoxic activity against tumor cells and provide an early host defense against the transformed cells.^2^ NKG2D is one of the most important NK cell-activating receptors, which is also expressed on a subset of CD8^+^ T cells, *γ*/*δ* T cells, NK1.1^+^ T cells and lymphokine-activated killer (LAK) cells.^3, 4, 5^ Ligands for NKG2D receptors (NKG2DLs) comprise major histocompatibility complex class I chain-related proteins A and B (MICA/B) and unique long 16 (UL16) binding proteins 1–6 (ULBP1–6).^6^ NKG2D–NKG2DL stimulation of NK cells leads to strong activation and tumor cell rejection.^7, 8, 9^ However, malignant cells decrease their surface expression of NKG2DLs through downregulation and/or internalization^10^ as well as the shedding of NKG2DL extracellular domains.^11^ The downregulation of NKG2DL prevents the detection of malignant cells by immune cells, although the underlying mechanisms remain unclear.

MicroRNAs (miRNAs) are short non-coding RNA molecules that usually repress gene expression by binding to the 3'-untranslated region (3'-UTR) of their target mRNAs. Increasing evidence indicates that miRNAs have important roles in tumor formation and immunogenicity.^12, 13, 14^ A group of miRNAs was predicted to target the mRNA of the NKG2DLs by the TargetScan database.^15^ Previous study found that miR-20a, miR-93, miR-106b, miR-373 and miR-520d could repress MICA and MICB expression by binding to the mRNA 3'-UTRs in human cancer cells (mainly HeLa, 293T, DU145 cells) and normal cells (human foreskin fibroblasts and human umbilical vein endothelial cells).^16^ Paula Codo *et al.*^17^ found that miR-20a, miR-93 and miR-106b contributed to the immune evasion of glioma cells by inhibiting MICA/B. These data led us to question whether miRNAs contribute to the post-transcriptional regulation of NKG2DL expression and how they influence the immunogenicity in the context of BC.

Histone deacetylase inhibitors (HDACis), as important reagents in epigenetic therapy, have shown promising effect in clinical trials for the treatment of human malignancies.^18^ Suberoylanilide hydroxamic acid (SAHA) and valproic acid (VPA) are potent HDACi drugs that have been approved by the United States Food and Drug Administration to treat cutaneous T-cell lymphoma and epilepsy, respectively. Both SAHA and VPA display antitumor activity *in vivo* with a favorable pharmacological profile and well-tolerated side effects.^19, 20^ In addition, HDACis might sensitize malignant cells to NK cell recognition depending on NKG2D–NKG2DL signaling.^21, 22^ These data suggest that HDACis may serve as a new and tumor-selective drug class by enhancing immune surveillance in the treatment of BC.

In the present study, we found that the high expression of MICB, which is an important NKG2DL, was an indicator of good prognosis in BC. Next, we characterized the important role of the miR-17–92 cluster in MICA/B and ULBP2 regulation and the functional impact of the miR-17–92 cluster on the BC immunogenicity. Furthermore, HDACis were found to enhance NK cell recognition in a miRNA-dependent manner.

## Results

### MICB is an indicator of good prognosis in BC

**Figure 1:**
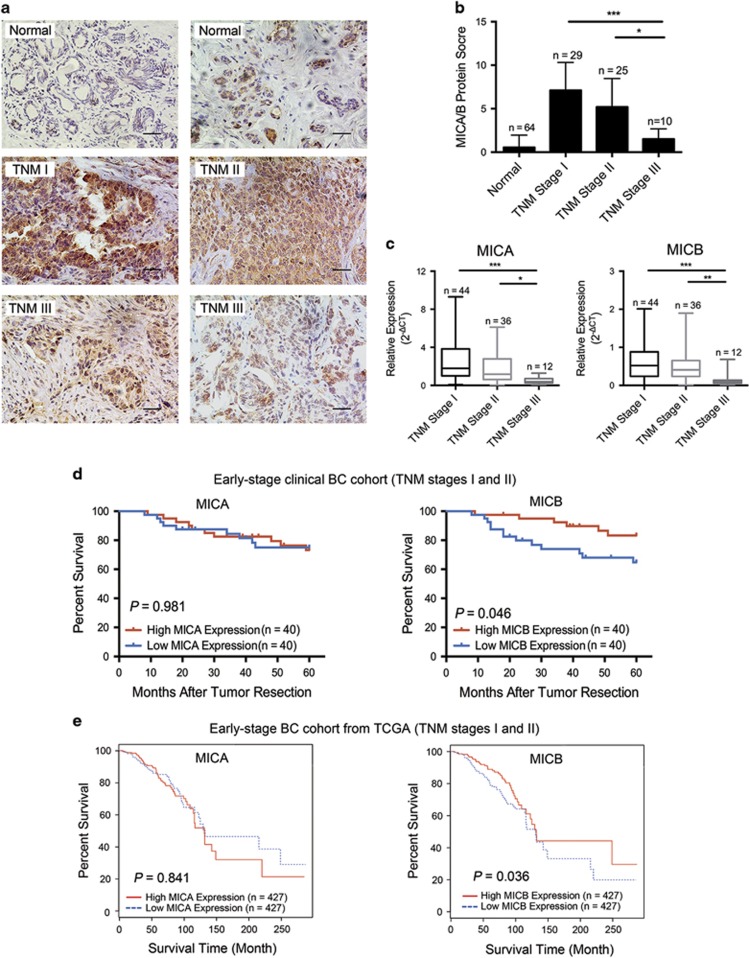
Clinical significance of the MICA/B expression profile in BC tissues. (**a**) Representative Immunohistochemistry (IHC) staining results for MICA/B expression in normal breast tissues and BC tissues with different TNM stages. (**b**) IHC scores of MICA/B in BC tissues were inversely associated with the TNM stage. (**c**) Quantitative PCR analysis. MICA (left) and MICB (right) mRNA expression levels were inversely associated with the TNM stage in BC tissues. (**d**) Kaplan–Meier survival curves of early-stage BC patients (TNM stages I and II) with different MICA (left) or MICB (right) expression levels (*N*=40 for each group). (**e**) Kaplan–Meier survival curves of early-stage BC cohort from TCGA (TNM stages I and II) with different MICA (left) or MICB (right) expression levels (*N*=427 for each group). *P<0.05, **P<0.01, *** *P*<0.001

MICA/B protein was rarely detected in the normal breast tissues of BC patients (84.4% showed negative MICA/B expression). However, in BC tissues, 92.2% showed positive MICA/B expression (Figures 1a and b). The MICA/B mRNA expression level was detected less in normal breast tissues than in paired BC tissues (Supplementary Figure 1a). Together, these results showed that the expression of MICA/B was higher in BC tissues than in normal breast tissues.

We found that the protein expression of MICA/B was significantly higher in BC cells at early stages (Tumor Node Metastasis (TNM) stages I and II) than in BC cells at advanced stages (TNM stage III) (Figures 1a and b). In addition, this inverse correlation was confirmed between MICA/B mRNA expression and the TNM stages by quantitative PCR analysis (Figure 1c). However, no correction was found between MICA/B expression and the WHO grade of BC (Supplementary Figure 1b). For ULBP1/2/3, no specific expression patterns were found for different TNM stages or WHO grades of BC (data not shown).

To determine the prognostic value of MICA/B expression in BC, 80 BC patients at the early stages of disease (TNM stages I and II) were divided into two groups according to the median value of MICA or MICB mRNA expression. Kaplan–Meier survival analysis was performed to evaluate the relationship between MICA or MICB expression and patient survival. The results indicated that patients with high expression of MICB had a longer overall survival compared with those with low expression of MICB. However, the results showed no significant difference in the overall survival between the two groups with different MICA expression (Figure 1d). Importantly, we found similar results in the early-stage BC cohort from The Cancer Genome Atlas (TCGA, TNM stages I and II) (*N*=855) with a long-term follow-up period (up to 20 years) (Figure 1e). Similar results were obtained if we included advanced-stage BC patients in the calculation above (Supplementary Figure 1c and 1d). Taken together, these results suggest that MICB might act as an important predictive factor in BC.

### Inverse correlation between the expression levels of endogenous NKG2DL-targeting miRNAs and NKG2DLs

The TargetScan database predicted numerous miRNAs with binding sites in the 3'-UTR of different NKG2DL members. Among them, miR-20a, miR-20b, miR-93 and miR-106b belong to the miR-17-92 cluster and its paralogs, which share a similar seed sequence. Members of the miR-17-92 cluster were reported to downregulate MICA/B in other carcinomas.^16, 17, 23^ Thus, we examined miR-20a, miR-20b, miR-93 and miR-106b as NKG2DL-targeting miRNAs in BC.

**Figure 2:**
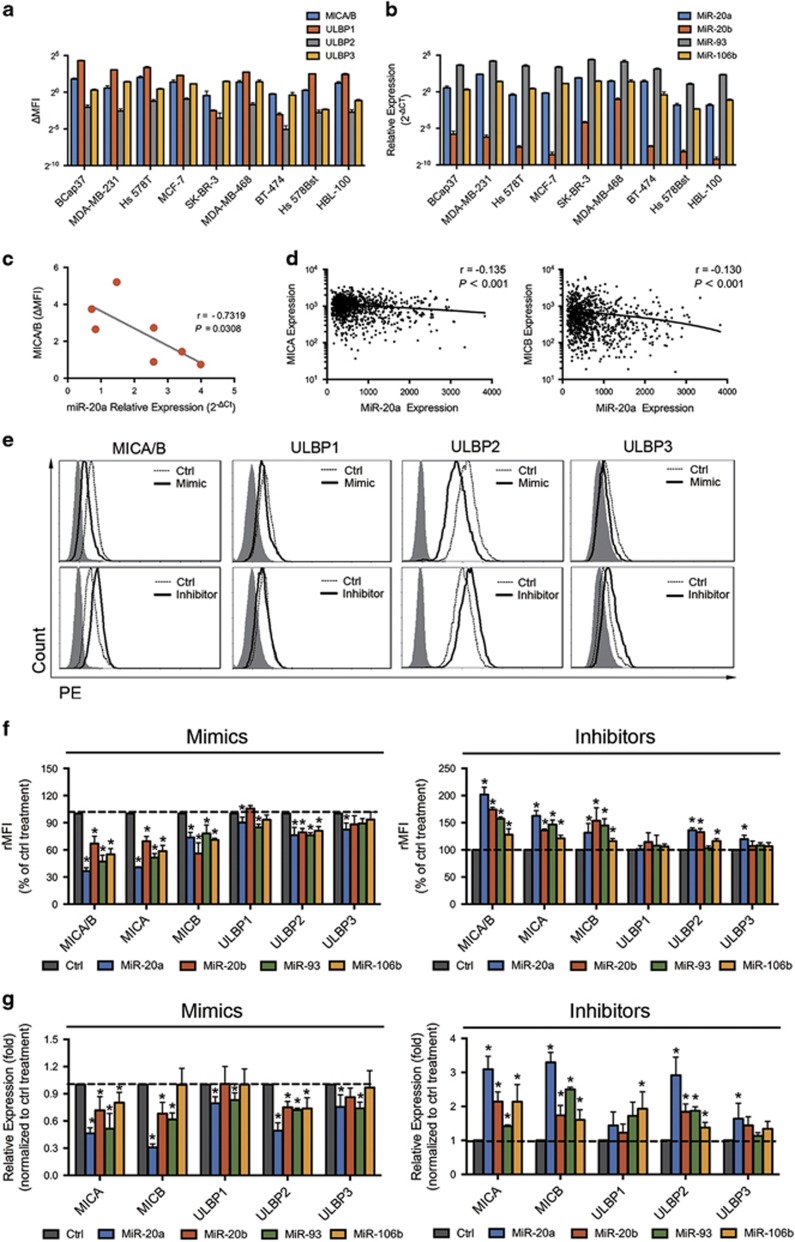
MicroRNAs specifically downregulate MICA/B and ULBP2 expression in BC cells. (**a**) The NKG2DL expression levels of seven human BC cell lines (BCap37, MDA-MB-231, Hs 578T, MCF-7, SK-BR-3, MDA-MB-468 and BT-474) and two normal breast cell lines (HBL-100 and Hs 578Bst) were assessed by flow cytometry analysis. (**b**) The miR-20a, miR-20b, miR-93 and miR-106b expression levels of the abovementioned cell lines were detected with quantitative PCR analysis. (**c**) Statistical analysis revealed a significant inverse correlation between the expression levels of miR-20a and MICA/B surface molecules in the seven BC cell lines. (**d**) Statistical analysis revealed an inverse correlation between the expression levels of miR-20a and MICA (left) or MICB (right) mRNA in TCGA's BC cohort (*N*=1042). (**e**), (**f**) and (**g**) BCap37 cells were exposed to control miRNAs (Ctrl), 50 nM mimics or inhibitors of specific miRNA for 24 h, respectively. The expression levels of NKG2DLs were detected 72 h after transfection. (**e**) Representative flow cytometry results showing that miR-20a inversely regulated the protein expression levels of MICA/B and ULBP2. (**f**) Flow cytometry analysis. The four tested miRNAs inversely regulated the protein expression levels of MICA/B and ULBP2. The relative MFIs (rMFIs) of NKG2DLs were calculated as follows: (ΔMFI of specific treatment/ΔMFI of control treatment) × 100%. (**g**) Quantitative PCR analysis. The four tested miRNAs inversely regulated the mRNA expression levels of MICA/B and ULBP2. Error bars represent the S.D. obtained from three independent experiments. *P<0.05

To assess the influence of endogenous miR-17-92 on NKG2DL expression in BC cells, seven human BC cell lines (BCap37, Hs 578T, MDA-MB-231, MDA-MB-468, BT-474, SK-BR-3 and MCF-7) and two human normal breast cell lines (HBL-100 and Hs 578Bst) were studied in parallel for their expression levels of NKG2DL surface proteins and the examined miRNAs. All the examined cell lines expressed NKG2DLs and the tested miRNAs at different levels (Figures 2a and b). In BC cell lines, statistical analysis revealed an inverse correlation between the expression levels of endogenous miR-20a and MICA/B protein expression (Figure 2c). Importantly, in the BC tissues from TCGA's cohort (*N*=1042), statistical analysis revealed an inverse correlation between the expression levels of endogenous miR-20a and MICA/B mRNA expression, which again pointed toward the involvement of the tested miRNAs in NKG2DL regulation (Figure 2d).

### MicroRNAs specifically downregulate MICA/B and ULBP2 expression in BC and normal breast cell lines

To assess the role of the tested miRNAs in the regulation of NKG2DL expression, BCap37 cells were transfected with control miRNAs (Ctrl), mimics or inhibitors of the tested miRNAs (Supplementary Figure 2a). Mimics of miR-20a, miR-20b, miR-93 and miR-106b decreased MICA/B and ULBP2 protein expression, whereas inhibitors of the tested miRNAs increased their expression in BCap37 cells (Figure 2e and f). However, the tested miRNAs showed limited influence on the expression of ULBP1/3. Among the four tested miRNAs, miR-20a showed the strongest influence on NKG2DL regulation in BCap37 cells (rMFI, mean±S.D., mimic *versus* Ctrl 47.2±5.7%, inhibitor *versus* Ctrl 202.0±10.1% for MICA/B, mimic *versus* Ctrl 76.1±2.3%, inhibitor *versus* Ctrl 136.4±3.3% for ULBP2, Figure 2f). In addition, these miRNA-mediated downregulations of NKG2DLs were typically associated with a decrease in related mRNA transcripts (Figure 2g). This finding indicated that the miRNA-mediated downregulation of NKG2DL expression was partially caused by enhancing degradation of related mRNA transcripts. We confirmed these results by assessing the effects of miR-20a and miR-93 on the BC cell line MDA-MB-231 and the normal breast cell line HBL-100 (Supplementary Figure 2b and c). Taken together, the tested miRNAs specifically downregulated MICA/B and ULBP2 expression in both BC and normal breast cell lines.

### MiR-20a/b directly bind to the MICA/B 3'-UTR

**Figure 3:**
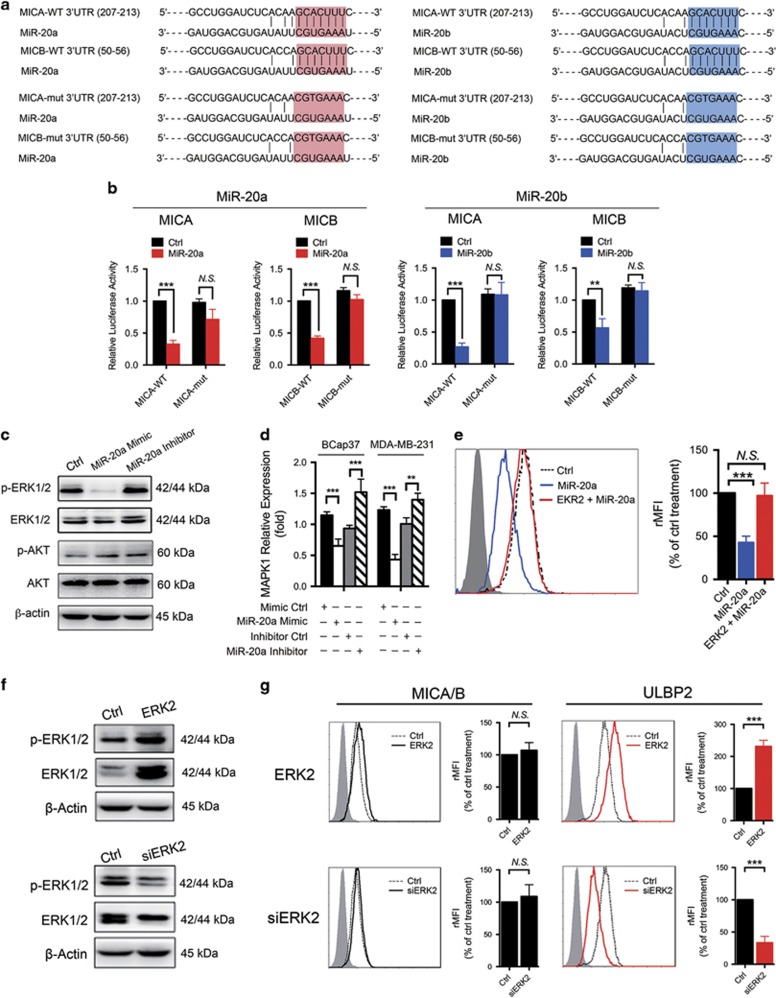
The mechanisms under miRNA-mediated MICA/B and ULBP2 downregulation. (**a**) Schematic representation of the miR-20a and miR-20b predicted binding sites in the 3'-UTRs of MICA/B mRNAs and 3'-UTR-mutated alignments. (**b**) Relative luciferase activity was assessed after transfecting the indicated reporter plasmids (MICA/B-WT 3'-UTR or MICA/B-mut 3'-UTR) into BCap37 cells with mimics of miR-20a, miR-20b or control miRNA (Ctrl). (**c**) and (**d**) BC cells were pre-exposed to lipo2000 only (Ctrl), a miR-20a mimic or inhibitor for 48 h. (**c**) Representative images of the western blotting assay. MiR-20a inversely regulated the expression of p-ERK1/2 (mainly p-ERK2) and ERK1/2 in BCap37 cells. (**d**) Quantitative PCR assay. MiR-20a inversely regulated the mRNA expression of ERK2 (MAPK1). (**e**) BCap37 cells were exposed to lipo2000 only (Ctrl), miR-20a mimic (miR-20a) or co-transfection of ERK2-pcDNA and miR-20a mimic (ERK2+miR-20a) for 48 h. Flow cytometry revealed that ERK2 overexpression could reverse miR-20a-mediated ULBP2 downregulation. (**f**) Representatives images of the western blotting assay. BCap37 cells were exposed to a pcDNA vector or control siRNA (Ctrl), ERK2-pcDNA (ERK) or ERK2-siRNA (siERK). (**g**) BCap37 cells were treated as shown in (**f**), and the flow cytometry analysis revealed that ERK2 effectively regulated the expression of ULBP2 but did not affected MICA/B. Error bars represent the S.D. obtained from three independent experiments. *P<0.05, **P<0.01, *** *P*<0.001

We next confirmed the specific interaction between the tested miRNAs and the mRNA of MICA/B. All tested miRNA have the same predicted binding sites in MICA or MICB mRNA. We used miR-20a and miR-20b as models. We generated four firefly luciferase reporter vectors: two containing the wild-type MICA or MICB 3'-UTR and the other two containing the MICA or MICB 3'-UTR with a mutated binding site (Figure 3a). Transfecting BCap37 cells with wild-type MICA or MICB in combination with the miR-20a or miR-20b mimic revealed a clear downregulation of luciferase activity compared with the control miRNA group (Ctrl). In contrast, the miR-20a or miR-20b mimic did not effectively reduce the luciferase activity of the MICA or MICB construct with a mutated binding site (Figure 3b). These results indicated that miR-20a and miR-20b directly targeted MICA/B mRNA at the predicted 3'-UTR-binding sites.

### MicroRNAs downregulate ULBP2 by inhibiting the MAPK/ERK signaling pathway

We found that ULBP2 had no predicted binding sites but was still effectively regulated by the tested miRNAs in different breast cell lines (Figures 2e–g and Supplementary Figure 2b and c). Both ERK and AKT are known to be involved in the regulation of ULBP2 expression.^24, 25, 26^ In addition, members of the miR-17-92 cluster target and inhibit the MAPK/ERK signaling pathway,^27, 28^ and miR-20a directly targets MAPK1 (ERK2) and inhibits its expression in BC cells (Wengong Si *et al.* unpublished data). For these reasons, the expression and activation status of the MAPK/ERK pathway were studied in untreated and miRNA-treated BCap37 cells. A western blotting assay showed that compared with the control treatment, the miR-20a mimic effectively inhibited the expression of p-ERK1/2 (mainly p-ERK2) and total ERK1/2. Moreover, the miR-20a inhibitor enhanced the expression of p-ERK1/2 and total ERK1/2, whereas neither the miR-20a mimic nor inhibitor affected AKT and p-AKT expression (Figure 3c). This miRNA-mediated downregulation of ERK2 was typically associated with a decrease in ERK2 (MAPK1) mRNA transcripts (Figure 3d). We confirmed these results by assessing the effects of miR-20b, miR-93 and miR-106b on ERK2 protein and mRNA expression in BCap37 cells. (Supplementary Figure d and e). To clarify the role of the MAPK/ERK signaling pathway in ULBP2 regulation, we silenced ERK2 expression by siRNA or increased constitutive ERK2 expression with ERK2-pcDNA transfection in BCap37 cells (Figure 3f). Flow cytometry analysis showed that the inhibition of ERK2 effectively downregulated the expression of ULBP2 but not MICA/B, whereas the overexpression of ERK2 increased the ULBP2 expression (Figure 3g). Moreover, ERK2 overexpression could reverse miR-20a-mediated ULBP2 downregulation in BCap37 cells (Figure 3e). These findings suggest that downregulated ULBP2 expression in miRNA-treated BC cells results from the MAPK/ERK signaling pathway inhibition.

### NK cell-mediated cytotoxicity is enhanced by silencing NKG2DL-targeting miRNAs *in vitro*

**Figure 4:**
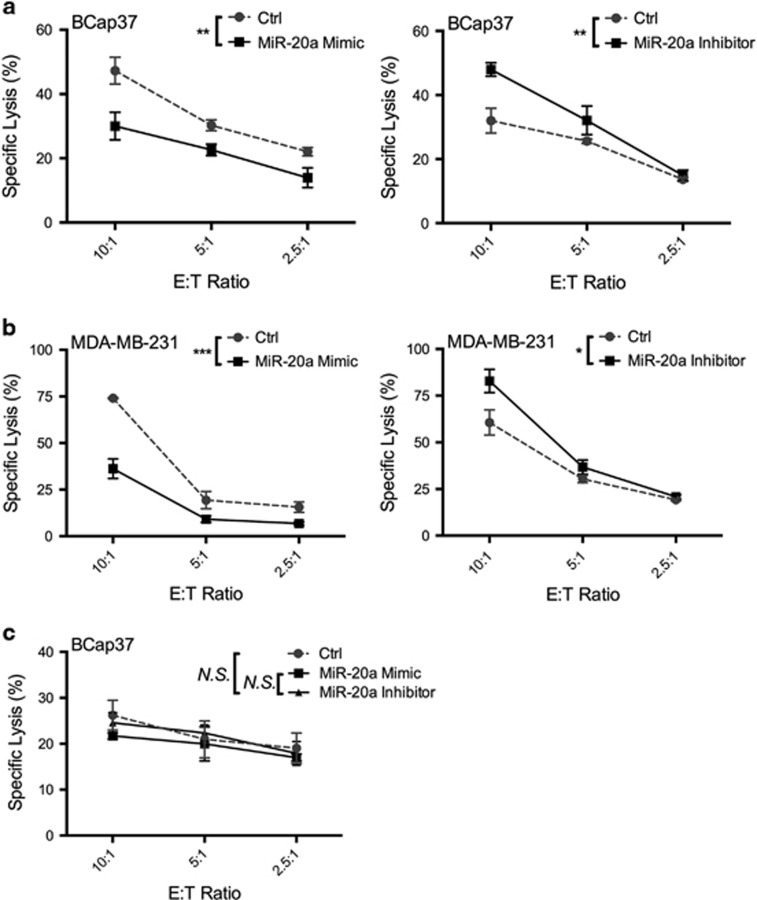
Silencing NKG2DL-targeting miRNAs in BC cells enhances NK cell-mediated killing *in vitro*. (**a**) BCap37 cells were exposed to control miRNAs (Ctrl), a miR-20a mimic or inhibitor for 24 h. The NK cells were pretreated with a control mAb for 1 h before the cytotoxicity assay. After 72 h of transfection, a 4-h cytotoxicity assay was performed using NK cells as effector cells. (**b**) MDA-MB-231 cells were treated and analyzed as in (**a**). (**c**) The NK cells were pretreated with an NKG2D-blocking antibody for 1 h before the cytotoxicity assay. Then, BCap37 cells were treated and analyzed as in (**a**). Error bars represent the S.D. obtained from three independent experiments. *P<0.05, **P<0.01, ***P<0.001

We next examined the effect of miRNA-mediated NKG2DL downregulation on NK cell recognition. BCap37 cells were transfected with control miRNA (Ctrl) or a miR-20a mimic or inhibitor. NK cells separated from the buffy coat of healthy donors were used in cytotoxicity assays at various effector-to-target (E:T) ratios. Transfection with a miR-20a mimic caused a remarkable reduction in the cytotoxicity activity at different E:T ratios. However, treatment with miR-20a inhibitor could effectively reverse these inhibitory effects (Figure 4a). These results implied that silencing NKG2DL-targeting miRNA could enhance NK cell-mediated cytotoxicity. Importantly, these differences between various treatments were abolished after NKG2D in NK cells was blocked with an anti-NKG2D monoclonal antibody (mAb) (Figure 4c). These results confirmed that the miRNA-mediated NK cell cytotoxicity was mediated through the NKG2D–NKG2DL pathway. The results were then verified using another human BC cell line (MDA-MB-231) as a target cell (Figure 4b) or LAK cells expanded from healthy donors as an effector cell (Supplementary Figure 2f).

### Silencing NKG2DL-targeting miRNA contributes to immune recognition *in vivo*

**Figure 5:**
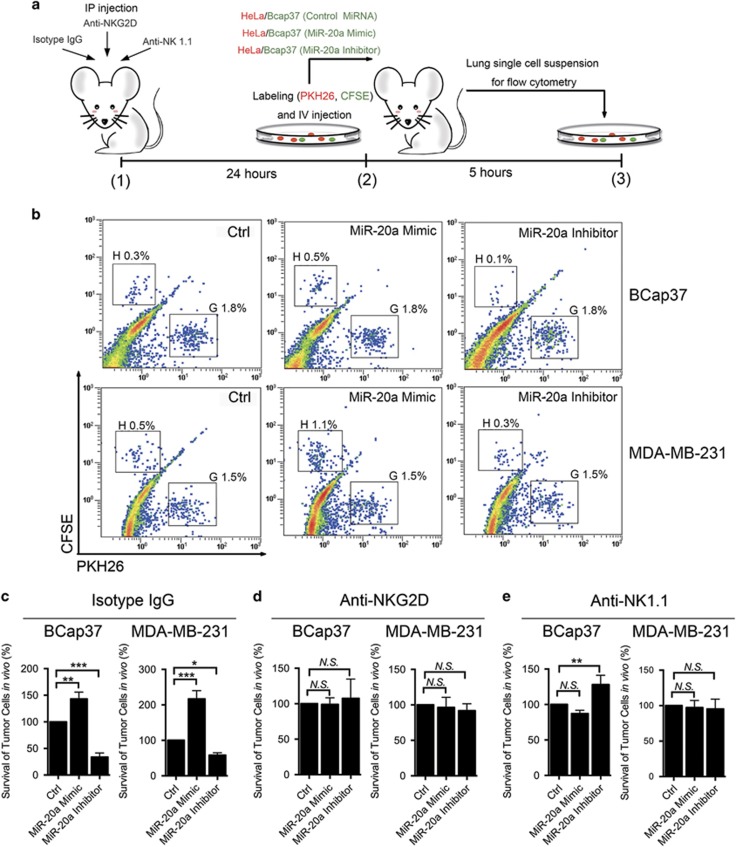
Silencing NKG2DL-targeting miRNAs in BC cells contributes to immune recognition *in vivo*. (**a**) A schematic diagram of the lung clearance assay of BCap37 cells. (**b**) Representatives flow cytometry results of the isotype IgG group. HeLa cells are shown in gate G, and tested cells (BCap37 or MDA-MB-231) with different treatments are shown in gate H. (**c**), (**d**) and (**e**) The relative ratios of tested cells to HeLa cells in the lungs were calculated in the isotype IgG group (**c**), anti-NKG2D group (**d**) and anti-NK1.1 group (**e**), respectively. For (**c**–**e**), the ratio of tested cells that treated with control miRNAs to HeLa cells was set to be 100%. Error bars represent the S.D. obtained from three independent experiments. *P<0.05, **P<0.01, ***P<0.001

To further investigate the effects of NKG2DL-targeting miRNAs on BC immunogenicity *in vivo*, we conducted a short-term lung clearance assay using C57BL/6 mice (Figure 5a). Although human NKG2DLs are different from mouse NKG2DLs,^29^ it is feasible to detect the activity of NKG2D-associated functions on human malignant cells in an immunocompetent mouse model.^30^ BCap37 cells treated with miR-20a mimic (which decreased NKG2DLs expression) were less susceptible to immune cell targeting. Thus, fewer cells were killed and more cells were detected in the harvested lungs than in those from the group transfected with control miRNA (Ctrl group) (fluorescence intensity (FI), mean±S.D. 143.1±10.53%). On the other hand, when NKG2DLs were increased with a miR-20a inhibitor, fewer labeled BCap37 cells were detected in the lungs compared with the Ctrl group (33.7±6.2%, Figure 5b top and 5c left). These results were confirmed using another BC cell line (MDA-MB-231) (mimic *versus* Ctrl 216.9±18.9%, inhibitor *versus* Ctrl 58.2±5.5%, Figure 5b bottom and 5c right). Importantly, the differences between different groups were abolished when NKG2Ds were blocked *in vivo* with an anti-NKG2D mAb in the BC cells (Figure 5d). The differences were reduced when NK cells were depleted with an anti-NK1.1 mAb in the BC cells. Interestingly, in the anti-NK1.1 group, BCap37 cells showed an increase of detection after treated with a miR-20a inhibitor, which required further observation. Taken together, these results confirmed that the miRNA-mediated regulation of immune recognition was through the NKG2D–NKG2DL pathway.

### HDAC inhibitors increase the expression of MICA/B and ULBP2 by inhibiting members of the miR-17-92 cluster

**Figure 6:**
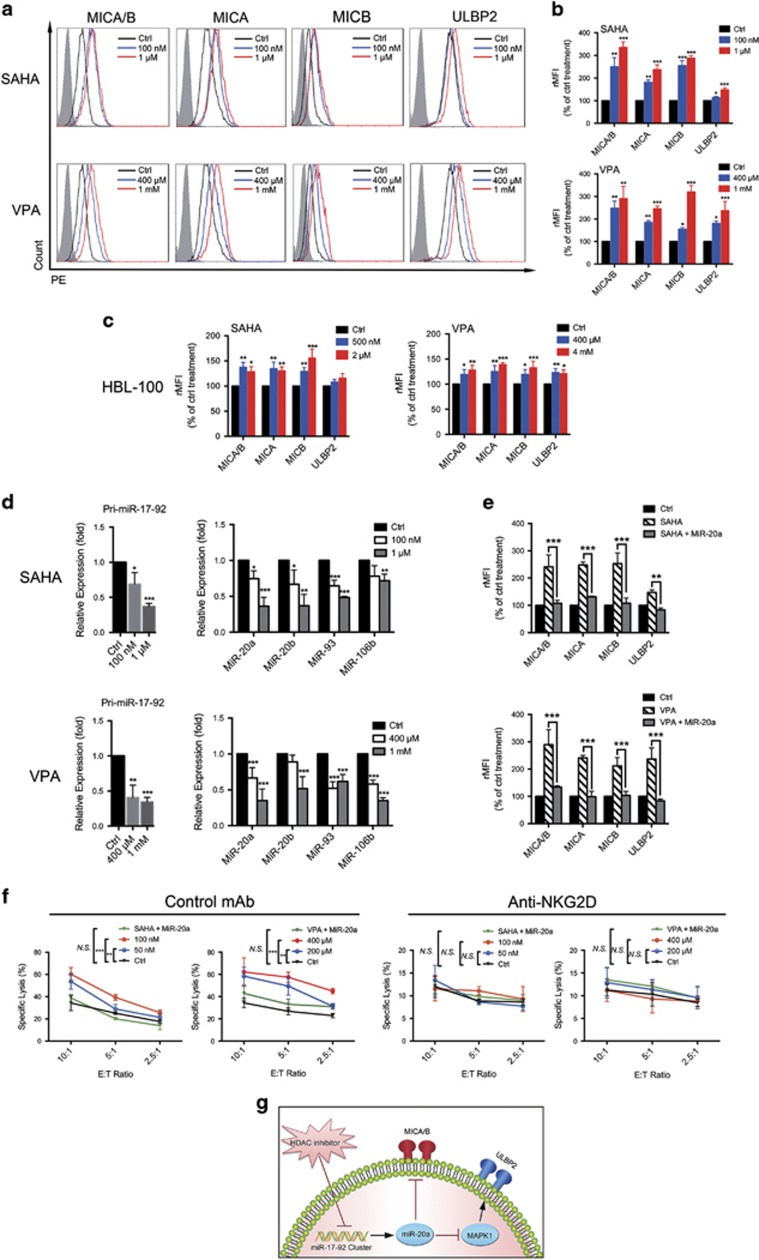
HDACis increase the expression of NKG2DLs by inhibiting members of the miR-17-92 cluster. (**a**) and (**b**) Flow cytometry analysis. HDACis SAHA and VPA upregulated the expression of MICA/B and ULBP2 in BCap37 cells in a dose-dependent manner. (**c**) Flow cytometry analysis. HDACis SAHA and VPA could not effectively upregulate the expression of MICA/B and ULBP2 in the human normal breast cell line HBL-100. (**d**) Quantitative PCR analysis. HDACis SAHA and VPA inhibited the expression levels of pri-miR-17-92, miR-20a, miR-20b, miR-93 and miR-106b in a dose-dependent manner. (**e**) BCap37 cells were treated with HDACis (100 nM SAHA or 400 *μ*M VPA) or HDACis together with miR-20a mimics(50 nM) for 48 h. Flow cytometry analysis revealed that miR-20a overexpression could reverse HDACi-mediated MICA/B and ULBP2 upregulation. (**f**) BCap37 cells were treated with different concentrations of HDACis or HDACis together with miR-20a mimics for 48 h, and then a 4 h cytotoxicity assay was performed using NK cells as effector cells. The NK cells were pretreated with control mAb or anti-NKG2D mAb 1 h before the cytotoxicity assay. (**g**) A schematic diagram of the mechanisms under HDACi-mediated NKG2DLs upregulation. Error bars represent the S.D. obtained from three independent experiments. *P<0.05, **P<0.01, *** *P*<0.001

The half maximal inhibitory concentrations (IC50) values of 48 h HDACis exposure in the tested cells were shown in Supplementary Figure 3a. To explore the impact of HDACis on BC cell immunogenicity, we exposed BCap37 cells to different concentrations of SAHA or VPA. HDACis dose-dependently increased MICA/B and ULBP2 expression (Figures 6a and b), whereas ULBP1/3 was not affected (data not shown). SAHA and VPA effectively increased MICA/B and ULBP2 expression, even at concentrations lower than 1/10 of the IC50 (100 nM for SAHA and 400 *μ*M for VPA), relative to control treatment (rMFI, mean±S.D. SAHA: 249.6±32.7% for MICA/B and 113.4±4.1% for ULBP2; VPA: 247.7±25.5% for MICA/B and 180.5±8.7% for ULBP2, Figure 6b). The results were then verified using another human BC cell line (MDA-MB-231) (Supplementary Figure 3b). Interestingly, the MICA/B and ULBP2 expression levels of the human normal breast cell line HBL-100 could not be effectively increased by HDACis, even at a concentration as high as the IC50 (rMFI, SAHA: 128.2±14.2% for MICA/B and 113.9±9.3% for ULBP2; VPA: 127.6±8.3% for MICA/B and 113.9±9.4% for ULBP2, Figure 6c). We found that this upregulation of NKG2DL expression was accompanied by the decreased expression of pri-miR-17-92, miR-20a, miR-20b, miR-93 and miR-106b in a dose-dependent manner (Figure 6d). The result indicated that HDACis might elevate the NKG2DLs expression in BC cell lines by inhibiting NKG2DL-targeting miRNAs. Moreover, a miR-20a mimic reversed HDACi-mediated MICA/B and ULBP2 upregulation in BCap37 cells (Figure 6e). To evaluate the effects of HDACi-mediated NKG2DL upregulation on NK cell recognition, we treated BCap37 or MDA-MB-231 cells with low concentrations of SAHA (50 nM or 100 nM) or VPA (200 *μ*M or 400 *μ*M) (Supplementary Figure 3a, c and d). Cells treated with HDACi became more sensitive to NK cell-specific cytotoxicity than cells cultured under standard conditions. In addition, these upregulation effects were reversed when cells were exposed to HDACis together with miR-20a mimics (Figure 6f left and Supplementary Figure 3e). Importantly, these differences between various treatments were abolished after NKG2Ds in NK cells was blocked with an anti-NKG2D mAb (Figure 6f right). Thus, by increasing NKG2DLs expression on BC cells, low concentrations of HDACis sensitized BC cells to NK cell-mediated cytotoxicity.

## Discussion

Previous studies have revealed that the immune system influences tumor development and clinical outcomes. NKG2DLs have an important role in cancer immunosurveillance and have clinicopathological significance.^31, 32, 33, 34, 35^ In BC, however, conflicting results have been reported. Some researchers implied that high expression of MICA/B resulted in a favorable outcome concerning the relapse-free period in early BC patients.^35^ However, higher MICA expression was found as an indicator of poor prognosis in BC.^36^ In addition, most previous studies were focused on the association of total MICA/B or MICA expression with clinical outcomes, whereas few considered the clinicopathological significance of MICB. In this study, we confirmed that MICA/B expression levels in BC tissues were inversely associated with TNM stage (Figures 1a–c). Importantly, we found that patients with high MICB expression showed longer overall survival than patients with low MICB expression. However, the different expression levels of MICA showed no significant influence on the survival of BC patients (Figures 1d and e). Together, these results suggest that MICB may act as a predictive factor in BC.

Members of the miR-17-92 cluster promote BC cell growth, migration and invasion.^37, 38^ Recently, miR-17-92 members were also reported to downregulate MICA/B protein expression in ovarian tumors,^23^ glioma^17^ and other tumors,^16^ contributing to their immune escape. In our study, we provided new evidence that members of the miR-17-92 cluster and its paralogs miR-20a, miR-20b, miR-93 and miR-106b specifically downregulated not only MICA/B but also ULBP2 expression in BC and normal breast cell lines (Figure 2). Importantly, in BC cells, miR-20b is newly reported in NKG2DL regulation. Unlike other studies,^17^ we found that this miRNA-mediated downregulation of MICA/B and ULBP2 was partly due to the enhanced degradation of related mRNA transcripts. The 3'-UTRs of MICA and MICB contain binding sites for the tested miRNAs, which was verified in BC cells using a dual-luciferase reporter gene assay (Figures 3a and b). Interestingly, we first found that ULBP2 had no predicted binding sites for the tested miRNAs but could still be effectively regulated in BC cells. This miRNA-mediated ULBP2 inhibition may result in miRNA-induced MAPK/ERK signaling pathway downregulation (Figures 3c–g and Supplementary Figure 2d and e). Members of the miR-17-92 cluster were previously reported to downregulate the expression of the MAPK/ERK signaling pathway.^27, 28^ In this study, western blot and quantitative PCR showed that the protein and mRNA levels of ERK2 (MAPK1) were inversely regulated by miR-20a, miR-20b, miR-93 and miR-106b. The MAPK/ERK pathway was reported to influence NKG2DLs expression in some other tumors.^39, 40^ For instance, the overexpression of constitutively active ERK (especially ERK1) in ARK cells was found increased MICA/B and ULBP2 expression.^25^ We found that ERK2 inhibition effectively downregulated the expression of ULBP2 but not MICA/B, whereas ERK2 overexpression increased the ULBP2 expression (Figures 3f and g). Our study further demonstrated the functional importance of miRNA-dependent regulation of NKG2DLs in BC cells using NK cell cytotoxicity assays (Figure 4). A short-term lung clearance model confirmed that silencing NKG2DL-targeting miRNA contributed to immune recognition *in vivo* (Figure 5). These findings emphasize the functional significance of the tested miRNAs in MICA/B and ULBP2 regulation.

However, miRNA candidates are based on modified nucleic acids and therefore still present delivery challenges in clinical applications.^41^ In contrast, SAHA and VPA are HDACis that have already been used in clinical scenarios. The present results revealed that treating BC cells with HDACis effectively sensitized tumor cells to NK cell recognition (Figure 6f). HDACis increase the expression of NKG2DLs in myeloma^25^ and colon cancer cells.^42^ In this study, we demonstrated that in BC cells, HDACis upregulated the expression of not only MICA/B but also ULBP2. This upregulation of NKG2DLs is typically accompanied by a decrease in the expression of miR-17-92 members (Figure 6d). The HDACi-induced overexpression of MICA/B and ULBP2 was reversed with a miR-20a mimic (Figure 6e). Previous study reported that HDACis butyrate, SAHA and VPA inhibited miR-17-92 transcription by reducing c-Myc expression.^42^ However, H Yang *et al.*^43^ proposed that SAHA downregulated the miR-17-92 cluster by abolishing tyrosine phosphorylation of STAT3 and decreased MCM7 transcription. Thus, the mechanisms for how HDACis downregulate the expression of miR-17-92 require further observation. In addition, we found that SAHA and VPA did not effectively elevate the NKG2DL expression of the normal breast cell line HBL-100 (Figure 6c). The basis for normal breast cell resistance to HDACis is not fully understood, although cancer cells with multiple defects cannot reverse the critical effects of HDACis similar to normal cells.^44, 45^ This resistance of normal breast cells presumably functions to avoid the autoimmunity caused by treatment with HDACis. Taken together, our study indicates that BC patients who receive HDACi treatment might show increased immune activation in addition to tumor cell inhibition.

The present study reports the following novel findings: (1) high MICB expression may be an indicator of a good prognosis in BC; (2) members of the miR-17-92 cluster inversely regulate ULBP2 and MICA/B in BC cells, which influences the immunogenicity of BC; (3) miRNAs downregulate the expression of MICA/B by targeting the mRNA 3'-UTR and downregulate ULBP2 by inhibiting the MAPK/ERK signaling pathway; (4) the HDACis SAHA and VPA may increase the expression of MICA/B and ULBP2 by inhibiting the miR-17-92 cluster; and (5) SAHA and VPA cannot effectively increase the expression of NKG2DLs in normal breast cells. In conclusion, this study is the first to comprehensively assess the miRNA-mediated NKG2DL regulation in the context of BC (Figure 6g). Our results indicate that targeting miRNAs with antisense inhibitors or by HDACis might therefore represent a novel approach to increasing the immunogenicity of BC.

## Materials and Methods

### Mice and cell lines

Male C57BL/6 (8–9 weeks old) mice were purchased from Vital River (Beijing, China). The human breast cell lines (BCap37, Hs 578T, MDA-MB-231, MDA-MB-468, BT-474, SK-BR-3, MCF-7, Hs 578Bst and HBL-100) were purchased from the cell bank of the Chinese Scientific Academy. BCap37, BT-474 and MCF-7 cells were cultured in Roswell Park Memorial Institute 1640 medium (Gibco, 31800105, Carlsbad, CA, USA) with 10% fetal bovine serum (FBS; Biological Industries, 04-0101-1, Cromwell, CT, USA). MDA-MB-231 and MDA-MB-468 cells were cultured in Leibovitz's L-15 medium (Gibco, 11415114) with 10% FBS. Hs 578T cells were cultured in Dulbecco's modified Eagle's medium (DMEM; Gibco, 12430047) supplemented with 0.01 mg/ml bovine insulin (Solarbio, I8040, Beijing, China) and 10% FBS. SK-BR-3 cells were cultured in McCoy's 5A medium (modified, Gibco, 16600082) with 10% FBS. Hs 578Bst cells were cultured in DMEM with 50 ng/ml epidermal growth factor (Gibco, PHG0311) and 15% FBS. HBL-100 cells were cultured in DMEM with 10% FBS. The cell culture medium was changed every 2–3 days, and the cells were passaged with 0.25% trypsin-EDTA (Gibco, 25200056) and grown to 90% confluence. The cultures were kept at 37 °C with 5% CO_2_ in a water-jacketed incubator (Thermo Scientific, Waltham, MA, USA).

### TCGA database

TCGA is available from the website of the Cancer Genomics Browser of the University of California, Santa Cruz (https://genome-cancer.ucsc.edu/). MICA and MICB mRNA expression and miRNA-sequencing level 3 data in BC patients were extracted from TCGA's data portal. Only patients with fully characterized tumors, TNM stage, overall survival, complete miRNA information were included. In total, data on 1096 BC patients with detailed miR-20a, MICA and MICB expression were collected from TCGA's data portal. Read counts for both mRNAs and miRNAs were used as input for survival analysis with the R package edgeR.^46, 47^ Kaplan–Meier plots for MICA or MICB expression in association with overall survival were calculated with the R program. Patients were split into high and low expression groups based on the median expression of MICA or MICB. For correlation analysis, the patients with values that were too low (<100) or too high (≥4000) for MICA/B mRNA or miR-20a expression were excluded (*N*=1042).

### 3'-UTR luciferase gene reporter assay

Dual-luciferase assays were performed using 1 × 10^4^ BCap37 cells per well in a 96-well plate (Corning/Costar, 3499, Acton, MA, USA). After the cells attached for 8 h, they were co-transfected with 50 ng of respective reporter constructs with either 50 nM of miRNA mimics or control miRNA. After 48 h, a Reporter Assay System Kit (Promega, 017319, Beijing, China) was used to measure the luciferase activity. There were three replicates for each transfectant. Firefly luciferase activity was normalized to constitutive Renilla luciferase activity.

### NK cell cytotoxicity assay

Respective sensitivity of BC cells to NK cells was determined using the CytoTox 96 cytotoxicity assay kit (Promega, 017317) according to the manufacturer's instructions. In brief, 1 × 10^4^ BCap37 or MDA-MB-231 target cells were seeded in triplicate at NK cell-to-target cell (E:T) ratios of 10:1, 5:1 and 2.5:1. An anti-NKG2D Ab (50 mg/ml, Novus, Littleton, USA, clone 149810) or control mAb (50 mg/ml, Novus) was added to experiments 1 h before co-culture. After 4 h of incubation, the supernatant was removed for analysis. The following formulas were used to calculate spontaneous and specific cytotoxicity: % specific release=(experimental release−effector spontaneous release−target spontaneous release)/(target maximum release−target spontaneous release) × 100%.

### Lung clearance assay

A short-term lung clearance assay was performed as previously described,^23, 30^ with some modifications to evaluate the NKG2DL-mediated immune effect *in vivo*. HeLa cells, which were not effectively recognized and killed by mouse NK cells, were used as internal negative controls. In brief, 36 male C57BL/6 mice were divided into three groups and were intraperitoneally injected with an IgG isotype control (300 *μ*g per mouse; Novus, Clone 20116, MAB004), an anti-mouse NKG2D mAb (300 *μ*g per mouse; Novus, Clone 191004, MAB1547) or an anti-NK1.1 Ab (300 *μ*g per mouse; Biolegend, Clone PK136, 108712, San Diego, CA, USA), respectively. Twenty-four hours later, HeLa cells were labeled with PKH26 (Invitrogen, MINI26-KT, Carlsbad, CA, USA), and various BCap37 cells (transfected with miR-20a mimic, miR-20a inhibitor, or control miRNAs) were labeled with CFSE (Invitrogen, C34554). Then, the stained BCap37 cells were mixed with the stained HeLa cells of each population at a density of 5 × 10^6^ cells in 1 ml PBS. A 0.4-ml aliquot of the cells was injected into the tail vein. Five hours later, the lungs were harvested to prepare single-cell suspensions, and flow cytometry analysis was performed. The relative ratio of tested cells to HeLa cells was calculated as the FI ratio of the tested cells to HeLa cells. Another human BC cell line (MDA-MB-231) was treated and analyzed as BCap37 cells.

Detailed methods are described in the Supplementary Materials, including immunohistochemical staining (IHC), transient transfection and drug treatment, clinical sample collection and NK cell isolation, RNA extraction and real-time quantitative PCR analysis, ligand expression analysis, Annexin V and propidium iodide staining analysis, flow cytometry analysis of cell cycle, western blotting assay, 3-(4,5-Dimethylthiazol-2-yl)-2,5-diphenyltetrazolium bromide assay, vector constructs, statistical analysis and ethics statement.
