# Supplementary material for: Silencing NKG2D ligand-targeting miRNAs enhances natural killer cell-mediated cytotoxicity in breast cancer
Source: Cell Death Dis. 2017 Apr 6;8(4):e2740–. doi: 10.1038/cddis.2017.158 (PMC5477582; doi:10.1038/cddis.2017.158)
Supplement: Supplementary Information 1 [file cddis2017158x1.docx]

**Supplementary Figure Legends**

**Supplementary Figure 1. Clinical significance of the MICA/B expression profile in BC tissues**. (**a**) The MICA (left) and MICB (right) mRNA expression levels in BC tissues and normal breast tissues from the same patient were assessed by quantitative PCR analysis (n = 32 for each group). (**b**) The relative expression levels of MICA (left) and MICB (right) mRNA in BC tissues with different WHO grades were assessed by quantitative PCR analysis. (**c**) Kaplan-Meier survival curves of BC patients with different MICA (left) or MICB (right) expression levels (n = 46 for each group). (**e**) Kaplan-Meier survival curves of TCGA’s BC cohort with different MICA (left) or MICB (right) expression levels (n = 548 for each group). **P* < 0.05, ***P* < 0.01, *** *P* < 0.001.

**Supplementary Figure 2. MicroRNAs specifically down-regulate the NKG2DL expression in BC cells.** (**a**) Quantitative PCR analysis. The miRNA mimics effectively increased the expression of the relevant miRNA, while the inhibitors decreased their expression. (**b**) and (**c**) Flow cytometry analysis. MiR-20a and miR-93 inversely regulated the expression levels of MICA/B and ULBP2 in both the human BC cell line MDA-MB-231 and normal breast cell line HBL-100. (**d**) and (**e**) BCap37 cells were pre-exposed to lipo2000 only (Ctrl), or mimics of miR-20b, miR-93 and miR-106b for 48 h. (**d**) Representatives images of the Western blotting assay. In BCap37 cells, miR-20b, miR-93 and miR-106b down-regulated the expression levels of p-ERK1/2 (mainly p-ERK2) and ERK1/2. (**e**) Quantitative PCR assay. MiR-20b, miR-93 and miR-106b down-regulated the mRNA expression of ERK2 (MAPK1). (**f**) BCap37 cells were exposed to control miRNAs (Ctrl), mimics or inhibitors of miR-20a for 24 h. After 72 h of transfection, a 4-h cytotoxicity assay was performed using lymphokine-activated killer (LAK) cells expanded from healthy donors as effector cells. Error bars represent the S.D. obtained from three independent experiments. **P* < 0.05, ***P* < 0.01, *** *P* < 0.001.

**Supplementary Figure 3.** **HDACis increase the expression of NKG2DLs** (**a**) MTT assay. The highest concentrations of HDACis that used in the ligand expression analysis caused less than 40% proliferation retardation in BC cells after 48 h of incubation (1 μM for SAHA and 1 mM for VPA, blue imaginary line). The highest concentrations of HDACis that used in the NK cell-cytotoxicity assay caused less than 10% proliferation retardation in BC cells after 48 h of incubation (100 nM for SAHA and 400 μM for VPA, red imaginary line). The IC50 values of 48 h HDACis exposure in the tested cells were listed in the table. (**b**) Flow cytometry analysis. HDACis SAHA and VPA up-regulated the expression of MICA/B and ULBP2 in MDA-MB-231 cells in a dose-dependent manner. (**c**) Annexin V and propidium iodide staining analysis. The highest concentrations of HDACis that used in the ligand expression analysis caused less than 6% cell apoptosis after 48 h of incubation (1μM for SAHA and 1 mM for VPA). The highest concentrations of HDACis that used in the NK cell-cytotoxicity assay caused less than 4% apoptosis (100 nM for SAHA and 400 μM for VPA). (**d**) Flow cytometry analysis of cell cycle. Low concentrations of SAHA and VPA had little influence on cell cycles of BCap37 and MDA-MB-231 after 48 h of incubation. (**e**) MDA-MB-231 cells were treated with different concentrations of HDACis or HDACis together with miR-20a for 48 h, and then a 4-h cytotoxicity assay was performed using NK cells as effector cells. The NK cells were pretreated with control mAb 1 h before the cytotoxicity assay. Error bars represent the S.D. obtained from three independent experiments. **P* < 0.05, ***P* < 0.01, *** *P* < 0.001.
