# Supplementary material for: Silencing NKG2D ligand-targeting miRNAs enhances natural killer cell-mediated cytotoxicity in breast cancer
Source: Cell Death Dis. 2017 Apr 6;8(4):e2740–. doi: 10.1038/cddis.2017.158 (PMC5477582; doi:10.1038/cddis.2017.158)
Supplement: Supplementary Information 2 [file cddis2017158x2.docx]

**Supplementary Materials and Methods**

**Immunohistochemical staining (IHC).** Antibodies specific for MICA/B (ab54413, Abcam, MA, USA) were used for the IHC analysis of paraffin-embedded sections from patients. The 4-μm sections were cut, deparaffinized and rehydrated. Endogenous peroxidases were blocked in 0.3% hydrogen-peroxide methanol for 20 min. Heat-induced antigen retrieval was conducted for 15 min in a microwave oven. Sections were incubated overnight with primary antibodies using predetermined optimal dilutions and incubation times. The slides were then incubated with the goat anti-rabbit IgG/HRP complex (ZSGB-BIO, PV-6001, Beijing, China) for 45 min at room temperature. After washing the slides two times in PBS, the sections were incubated with horseradish peroxidase-conjugated streptavidin (DAB kit, ZSGB-BIO, ZLI-9019, China) for 30 min and then washed twice in PBS. The positive results were visualized with 3,3’-diaminobenzidine (DAB) (DAB kit, ZSGB-BIO, ZLI-9019, China).

The results of IHC were analyzed by examination and photography under microscopy with × 400 magnification. All of the slices were evaluated by two pathologists without knowledge of the clinical outcome. The percentage of immune-positive cells and the staining intensities were evaluated in each sample. The percentage of immune-positive cells was graded on a scale from 0 to 4, where no staining was scored as 0; 1-10% of cells stained was scored as 1; 11-50% was scored as 2; 51-80% was scored as 3; and 81-100% was scored as 4. The staining intensities were graded from 0 to 3, where 0 was defined as negative; 1 as weak; 2 as moderate; and 3 as strong. The MICA/B protein score was calculated as the product of intensity and percentage scores, which ranged from 0 to 12. .The MICA/B expression level was characterized as low or high based on the median total score ^1^.

**Transient transfection and drug treatment.** Human breast cell lines were seeded at an adequate density in 6-cm or 10-cm (430167 or 430196, Corning, NY, USA) cell culture dishes. After the cells had attached, 50 nM control miRNAs (RiboBio Co. Ltd, miRNA mimic control, miR01101; miRNA inhibitor control, miR02101, Guangdong, China), mimics or inhibitors of certain miRNAs (Ribobio, Supplementary Table 2 and 3) were transfected together with the Lipofectamine™ 2000 transfection reagent (Invitrogen, 11668019, USA). All of the steps were performed according to the manufacturer’s instructions. After 24 h of transfection, the medium was replaced. Then, siRNA of MAPK1 and negative control siRNA were purchased from GenPharma (Supplementary Table 5). Suberoylanilide hydroxamic acid (SAHA) was purchased from Selleck Chemicals (MK0683, Houston, USA), stored at -20°C, and dissolved in dimethylsulfoxide (DMSO, Sinopharm Chemical Regent Co., Shanghai, China) to make a 5 mM stock. Valproic acid (VPA) was purchased from Sigma (P4543, USA), stored at 4°C, and dissolved in PBS to make a 10 mM stock.

**Clinical sample collection and NK cell isolation.** Paraffin-embedded sections of BC tissues were obtained from 92 BC patients. Sixty-four of the patients also provided related normal breast tissues. The patient cohort was comprised of non-metastasized BC patients who were primarily treated with surgery between 2010 and 2011. All tissues were collected during surgery. Patients with TNM stage IV, basal cell carcinoma, cervical carcinoma *in situ,* or a prior history of cancer were excluded. The following data were obtained for the included patients: age, tumor WHO grade, TNM stage, estrogen receptor (ER) status, progesterone receptor (PR) status and p53 protein status (Supplementary Table 1). All parameters were determined according to the current pathology standards. The buffy coats from healthy donors were preserved at 4°C and used within 6 h. All of the clinical samples were obtained from the First Affiliated Hospital of Zhejiang University School of Medicine.

For NK cell isolation, peripheral blood mononuclear cells (PBMCs) were prepared from the buffy coats using Ficoll-Paque (TBDscience, HY2015, Tianjin, China) and washed twice with PBS (Gibco, 10010023). NK cells were negatively isolated from PBMCs using the Human NK Cell Enrichment Set-DM (BD Bioscience, San Jose, USA) according to the manufacturer’s instructions, and the percentage of NK cells was confirmed by flow cytometry. The isolated cells were used for the following experiment when the percentage of CD3^-^CD56^+^ was higher than 90%.

**RNA extraction and real-time quantitative PCR analysis.** Total RNA from BC cell lines was extracted using RNAiso Plus (TaKaRa, 9109, Kusatsu, Japan) following the manufacturer’s instructions. Total RNA from the paraffin-embedded sections of patients was extracted using the RNeasy^®^FFPE Kit (Invitrogen, 73504, Carlsbad, CA).

For mRNA quantitative PCR analysis, the total RNA was then reverse transcribed into cDNA (TaKaRa, R0037A) by random primers. Gene expression was measured using a LightCycler 480II system (Roche Diagnostics, Basel, Switzerland) with SYBR Premix EX Tag kit (TaKaRa, RR420A) and specific primers (Supplementary Table 4). The miRNA quantification was determined by Bulge-loop^TM^ miRNA qRT-PCR Primer Set (one RT primer and a pair of qPCR primers for each set) specific for miR-20a, miR-20b, miR-93 or miR106b and U6, designed by RiboBio (Supplementary Table 2). Relative RNA expression was calculated with the 2 -ΔCt or 2 –ΔΔCt method after normalizing the expression levels of tested mRNA to HPRT1 mRNA and tested miRNA to U6 miRNA.

**Ligand expression analysis.** The following antibodies were purchased from R&D systems (Minneapolis, MN, USA): MICA/B-PE (clone 159207, FAB13001P), MICA-PE (clone 159227, AB1300P), MICB-PE (FAB1599P), ULBP1-PE (clone 170818, FAB1380P), ULBP2/5/6-PE (clone 165903, FAB1298P), and ULBP3-PE (clone 166510, FAB1517P). CD3-PE (clone HIT3a, 300308) and CD56-APC (clone HCD56, 318309) were purchased from Biolegend (San Diego, CA). Cells were collected and washed with PBS twice and incubated with antibodies for 25 min at 4°C in the dark, followed by flow cytometry analysis. Flow cytometry was performed using a BD FACSCalibur™ flow cytometry system. The increase in mean fluorescence intensity (ΔMFI) was calculated as follows: (MFI with specific mAb – MFI with isotype control) / MFI with isotype control. The relative MFI (rMFI) value was calculated to compare the difference between the ΔMFI of a specific treatment and control as follows: ΔMFI of specific treatment / ΔMFI of control treatment × 100%.

**Annexin V and propidium iodide staining analysis.** Cells with different treatments were collected and subjected to Annexin V and propidium iodide staining using Annexin V-FITC/PI apoptosis kit (Multi Science, AP101, China) according to the manufacturer’s protocol. After staining, flow cytometry was performed to quantify apoptotic cells.

**Flow cytometry analysis of cell cycle.** Cells were fixed in −20 °C absolute ethanol for 4 h and resuspended in 3 ml of PBS. After incubating at 37 ^o^C for 30 min, the cells were treated with DNA staining solution (Multi Science, CCS012) at room temperature for 10 min in the dark. A total of 10,000 cells were analyzed by flow cytometry.

**Western blotting assay.** Antibodies specific to phospho-ERK1/2 (4379), ERK1/2 (9102), AKT (4596), phospho-AKT (Ser473, 5012) and β-Actin (8457) were purchased from Cell Signaling Technology (CST, Beverly, MA). The cell lysates were collected with radioimmunoprecipitation assay (RIPA) lysis buffer (Beyotime Biotechnology, P0013B) and 1 mM phenylmethanesulfonyl fluoride (PMSF; Beyotime Biotechnology, ST506). The protein concentration was assessed using a BCA assay (BCA Protein Assay Kit, Beyotime, P0012). Denatured protein lysates (50 mg, 70°C for 10 min) were separated on 10% SDS-polyacrylamide gels and transferred to PVDF membranes (0.2 mm, Millipore, ISEQ00010, Darmstadt, Germany). Western blotting was performed according to the manufacturer’s instructions. Membranes were blocked in Tris-buffered saline containing 0.05% Tween-20 (Sangon Biotech, A100777) and 5% non-fat dry milk for 1 h. After blocking, the membranes were probed overnight at 4°C with primary antibodies. Then, the blots were incubated for 2 h at room temperature in a 1:10,000 dilution of HRP goat anti-rabbit IgG antibody (Abcam, ab6721) after 3 washes with PBST. After extensive washing, antibody detection was accomplished with a sensitive substrate (Immun-Star^TM^ Western C^TM^ Kit, Bio-Rad, #170-5070).

**3-(4, 5-Dimethylthiazol-2-yl)-2, 5-diphenyltetrazolium bromide assay.** Cells were harvested and resuspended to a final concentration of 10,000 cells/ml. Aliquots of the cell suspension were evenly distributed into 96-well tissue culture plates. After one night of incubation, the designated columns were treated with HDACis. Four hours prior to the end of time point, MTT solution was added. The medium containing MTT was replaced with 150 μL of DMSO in each well to dissolve the formazan crystals after 4 h-incubation. The absorbance in individual wells was determined at 560 nM using a microplate reader (Bio-Rad, Sunnyvale, CA).

**Vector constructs.** Expression vectors encoding MAPK1 were constructed by cloning the open reading frames into the pcDNA 3.1 vector (Invitrogen) between the HindIII and EcoRI sites for expression driven by the CMV promoter (ERK2-pcDNA). The wild-type or mutant 3’-UTR of MICA or MICB mRNA was cloned into the psiCHECKTM-2 vector (Promega).

**Statistical analysis.** Statistical analyses were performed with GraphPad Prism software (GraphPad, San Diego, CA, USA). Briefly, the data are presented as mean ± standard deviation (S.D.) of three independent experiments. Two-way analysis of variance (ANOVA) followed by Bonferroni’s posttest was used to compare the means of groups influenced by two independent factors, whereas one-way ANOVA followed by Tukey’s posttest was used to compare the means of three independent groups. Student’s *t*-test followed by Welch’s correction was used to compare the means of two independent groups. The correlation between two factors was evaluated by correlation analysis, and Spearman correlation coefficients were calculated to estimate the correlations. Statistical evaluation of IHC scores or mRNA expression levels in clinical BC and normal tissues was performed with the Kruskal-Wallis test with Dunn’s correction for multiple testing. The Kaplan-Meier survival function was calculated and compared with a log-rank test to assess the differences in BC samples. *P* values < 0.05 were considered statistically significant.

**Ethics statement.** All studies that involved human participants were approved by the Ethics Committee of Zhejiang University School of Medicine, Hangzhou, China, and the study methodology was carried out in accordance with the approved guidelines. Informed consent was obtained from each subject before participating in the study, according to the Declaration of Helsinki. All of the animal experiments in this study were approved by the Zhejiang University Animal Care Committee, Hangzhou, China. All animal manipulations were carried out according to the National Institutes of Health Guidelines for the Care and Use of Laboratory Animals (NIH Publication, 8^th^ edition) as revised in 2012. The mice were sacrificed using carbon dioxide inhalation. All surgeries were conducted under sodium pentobarbital anesthesia, and all efforts were made by the attending skilled technician to minimize suffering.

**References:**

1. Fang L, Gong J, Wang Y, Liu R, Li Z, Wang Z, Zhang Y, Zhang C, Song C, Yang A, Ting JP, Jin B, Chen L. MICA/B expression is inhibited by unfolded protein response and associated with poor prognosis in human hepatocellular carcinoma. J Exp Clin Cancer Res 2014; 33:76.
