# Supplementary material for: Silencing NKG2D ligand-targeting miRNAs enhances natural killer cell-mediated cytotoxicity in breast cancer
Source: Cell Death Dis. 2017 Apr 6;8(4):e2740–. doi: 10.1038/cddis.2017.158 (PMC5477582; doi:10.1038/cddis.2017.158)
Supplement: Supplementary Information 3 [file cddis2017158x3.docx]

Supplementary Table 1: Patient Information

|  | | **N** | **%** |
| --- | --- | --- | --- |
| **Age** | |  |  |
| < 50 | | 41 | 44.6% |
| ≥ 50 | | 51 | 55.4% |
| **Grade** |  |  |  |
| I | | 11 | 12.0% |
| II | | 51 | 55.4% |
| III | | 30 | 32.6% |
| **TNM stage** | |  |  |
| I | | 44 | 47.8% |
| II | | 36 | 39.1% |
| III | | 12 | 13.0% |
| **ER status** | |  |  |
| Negative | | 41 | 44.6% |
| Positive | | 51 | 55.4% |
| **PR status** | |  |  |
| Negative | | 48 | 52.2% |
| Positive | | 44 | 47.8% |
| **p53 status** | |  |  |
| Negative | | 19 | 20.7% |
| Low | | 40 | 43.5% |
| High | | 33 | 35.9% |

Abbreviations: N, number of patients; %, percentage; TMN, Tumor Node Metastasis; p53, p53 protein positivity; ER, estrogen receptor; PR, progesterone receptor.

Supplementary Table 2: miRBase Accession for tested miRNA

| Transcript | miRBase Accession |
| --- | --- |
| hsa-mir-20a | MIMAT0000076 |
| hsa-mir-20b | MIMAT0001519 |
| hsa-mir-93 | MIMAT0000093 |
| hsa-mir-106b | MIMAT0000734 |

Supplementary Table 3: miRNA mimics and inhibitors

| **Name** | **Sequence (5’-3’)** |
| --- | --- |
| **hsa-mir-20a** |  |
| Mimic | GUAGCACUAAAGUGCUUAUAGUGCAGGUAGUGUUUAGUUAUCUACUGCAUUAUGAGCACUUAAAGUACUGC |
| Inhibitor | GCAGUACUUUAAGUGCUCAUAAUGCAGUAGAUAACUAAACACUACCUGCACUAUAAGCACUUUAGUGCUAC |
| **hsa-mir-20b** |  |
| Mimic | AGUACCAAAGUGCUCAUAGUGCAGGUAGUUUUGGCAUGACUCUACUGUAGUAUGGGCACUUCCAGUACU |
| Inhibitor | AGUACUGGAAGUGCCCAUACUACAGUAGAGUCAUGCCAAAACUACCUGCACUAUGAGCACUUUGGUACU |
| **hsa-mir-93** |  |
| Mimic | CUGGGGGCUCCAAAGUGCUGUUCGUGCAGGUAGUGUGAUUACCCAACCUACUGCUGAGCUAGCACUUCCCGAGCCCCCGG |
| Inhibitor | CCGGGGGCUCGGGAAGUGCUAGCUCAGCAGUAGGUUGGGUAAUCACACUACCUGCACGAACAGCACUUUGGAGCCCCCAG |
| **hsa-mir-106b** |  |
| Mimic | CCUGCCGGGGCUAAAGUGCUGACAGUGCAGAUAGUGGUCCUCUCCGUGCUACCGCACUGUGGGUACUUGCUGCUCCAGCAGG |
| Inhibitor | CCUGCUGGAGCAGCAAGUACCCACAGUGCGGUAGCACGGAGAGGACCACUAUCUGCACUGUCAGCACUUUAGCCCCGGCAGG |

Supplementary Table 4: Primers for mRNA quantitative PCR

| **Primer** | **Sequence (5’-3’)** |
| --- | --- |
| MICA-F | ACAATGCCCCAGTCCTCCAGA |
| MICA-R | ATTTTAGATATCGCCGTAGTTCCT |
| MICB-F | TGAGCCCCACAGTCTTCGTTAC |
| MICB-R | TGCCCTGCGTTTCTGCCTGTCATA |
| ULBP1-F | TGCAGGCCAGGATGTCTTGT |
| ULBP1-R | CATCCCTGTTCTTCTCCCACTTC |
| ULBP2-F | CAGAGCAACTGCGTGACATT |
| ULBP2-R | GGCCACAACCTTGTCATTCT |
| ULBP3-F | GGATTTCACACCCAGTGGAC |
| ULBP3-R | GCCTCTTCTTCCTGTGCATC |
| Pri-miR-17-92-F | CAGTAAAGGTAAGGAGAGCTCAATCTG |
| Pri-miR-17-92-R | CAGTAAAGGTAAGGAGAGCTCAATCTG |
| GAPDH-F | TCTCCTCTGACTTCAACAGCGAC |
| GAPDH-R | CCCTGTTGCTGTAGCCAAATTC |
| HPRT1-F | TGACACTGGCAAAACAATGCA |
| HPRT1-R | GGTCCTTTTCACCAGCAAGCT |

Supplementary Table 5: Sequence for siRNAs

| siRNA | Sequence (5’-3’) |
| --- | --- |
| siMAPK1#1(sense) | CAUGGUAGUCACUAACAUAdTdT |
| siMAPK1#1(antisense) | UAUGUUAGUGACUACCAUGdAdT |
| siMAPK1# 2 (sense) | UUAUUAUACAAUUCAGAUCTT |
| siMAPK1#2 (antisense) | UUAUUAUACAAUUCAGAUCTT |
