# Supplementary figures and images for: Silencing NKG2D ligand-targeting miRNAs enhances natural killer cell-mediated cytotoxicity in breast cancer
Source: Cell Death Dis. 2017 Apr 6;8(4):e2740–. doi: 10.1038/cddis.2017.158 (PMC5477582; doi:10.1038/cddis.2017.158)

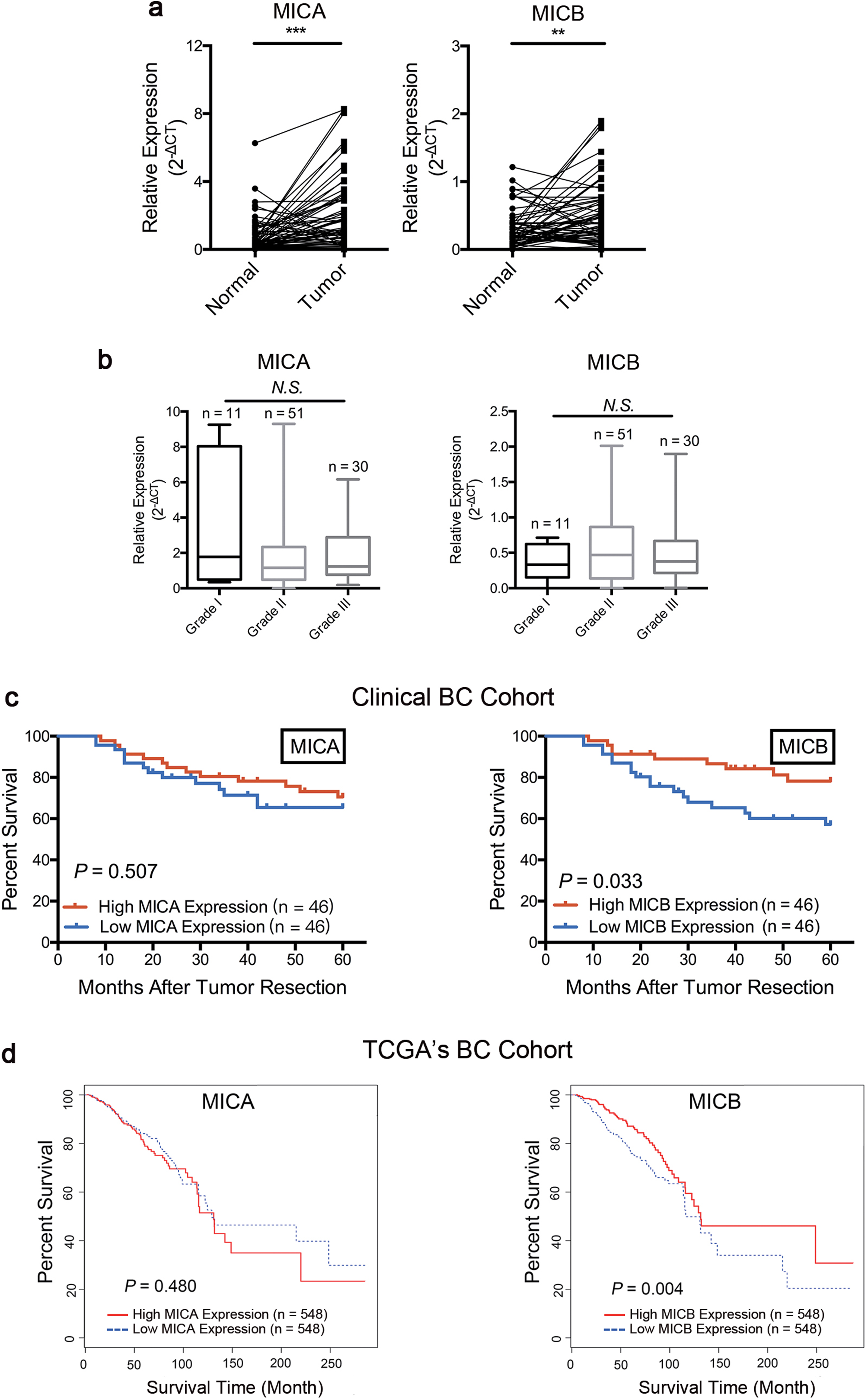

Supplement: Supplementary Figure 1 [file cddis2017158x4.tif]

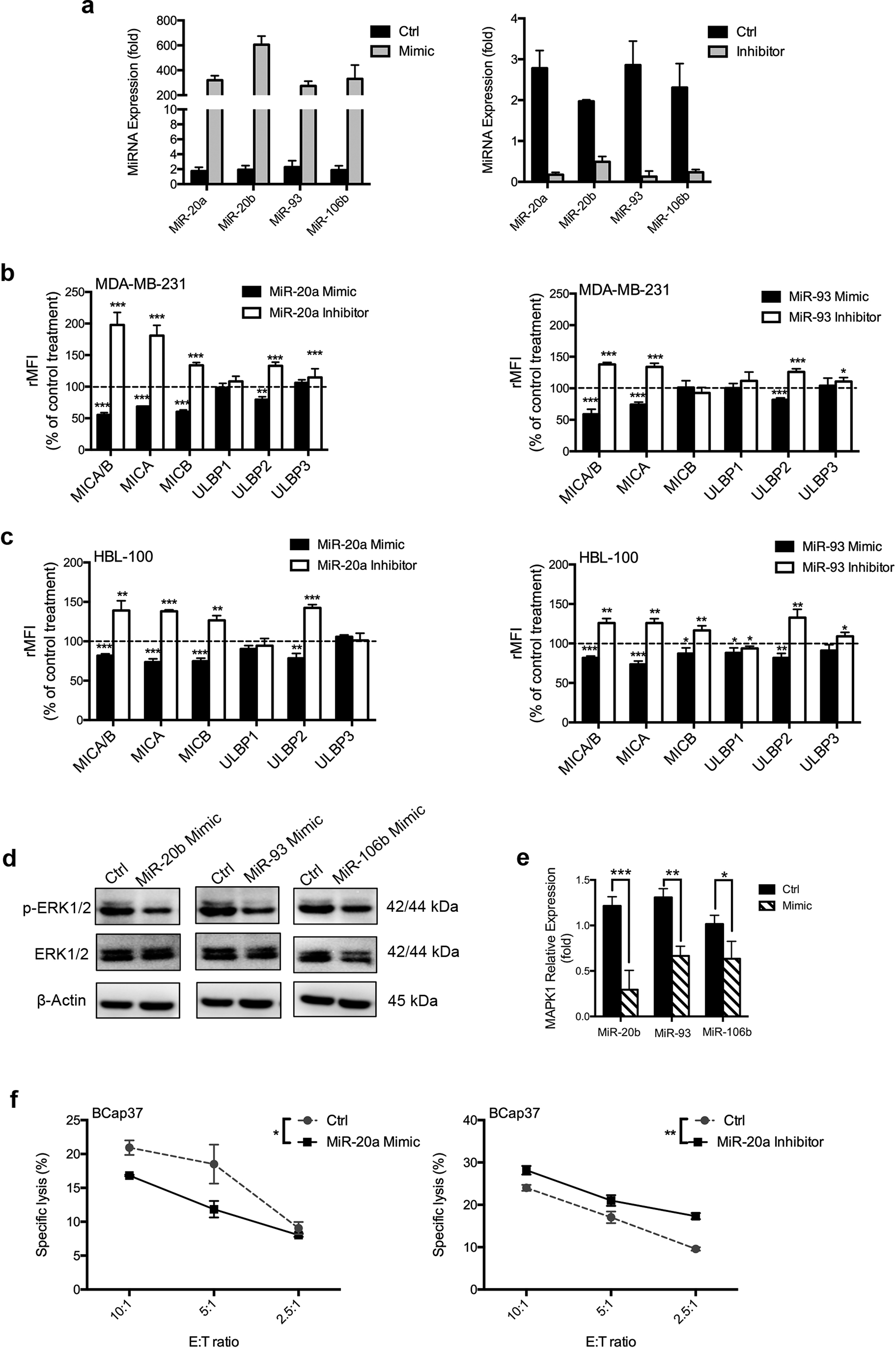

Supplement: Supplementary Figure 2 [file cddis2017158x5.tif]

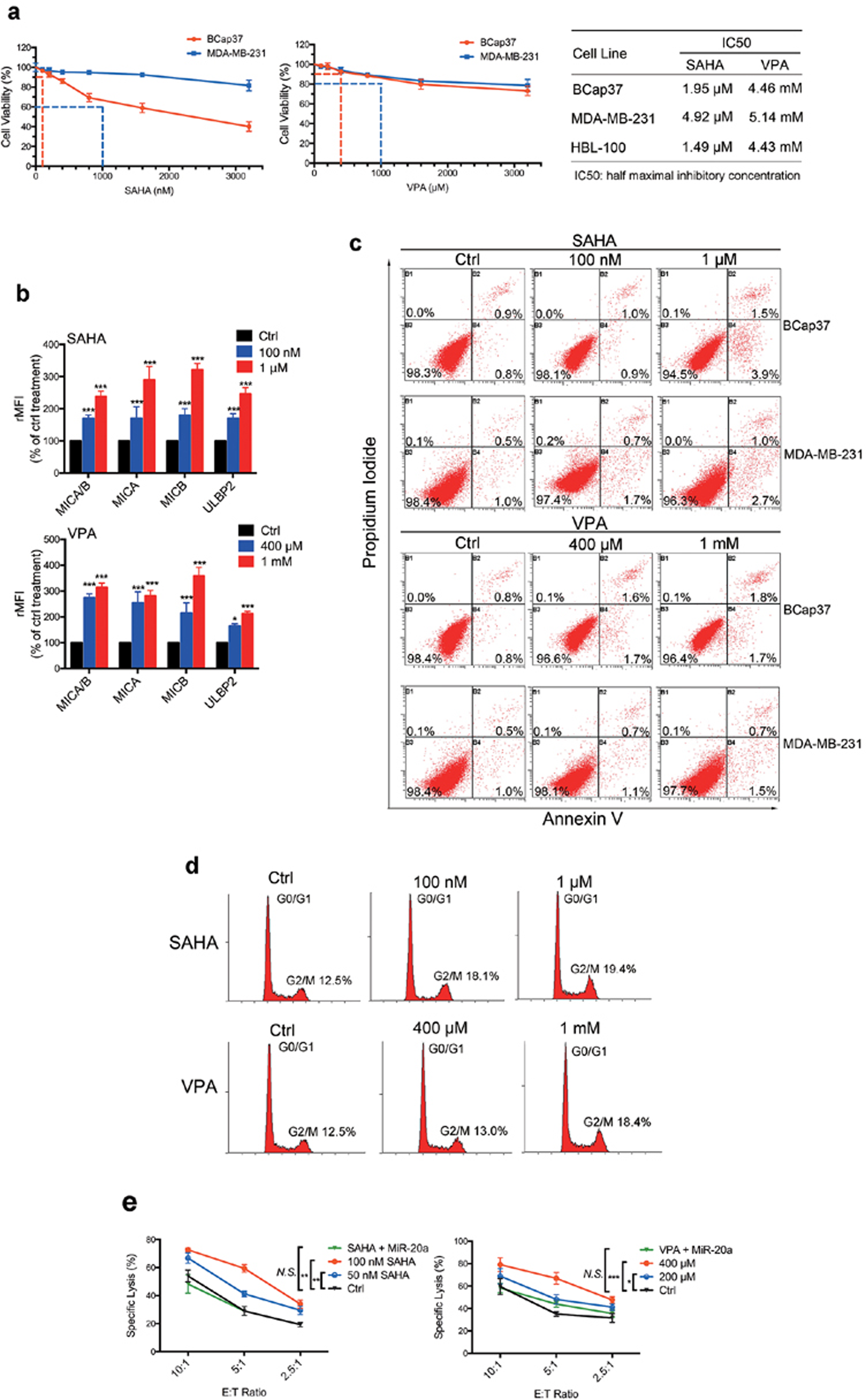

Supplement: Supplementary Figure 3 [file cddis2017158x6.tif]
